# Polyketide-Derived Antimicrobial Metabolites from *Epicoccum sorghinum*: Effects of Fermentation Conditions and Non-Thermal Atmospheric Pressure Plasma (NTAPP)

**DOI:** 10.4014/jmb.2606.06052

**Published:** 2026-10-13

**Authors:** Ching-Chia Chang, Bai-Wei Peng, Annamária Kincses, Chi-Ying Li, Hua-Han Chen, Hsueh-Wei Chang, Judit Hohmann, Fang-Rong Chang

**Affiliations:** 1 Graduate Institute of Natural Products, College of Pharmacy, Kaohsiung Medical University, Kaohsiung, Taiwan; 2 Pharmacognosy, Department of Pharmaceutical Biosciences, Biomedical Centre, Uppsala, Sweden; 3 Institute of Pharmacognosy, University of Szeged, Szeged, Hungary; 4 School of Pharmacy, Tzu Chi University, Hualien, Taiwan; 5 Department of Food Science, National Penghu University of Science and Technology, Makung City, Penghu Hsien, Taiwan; 6 Cancer Center, Kaohsiung Medical University Hospital, Kaohsiung Medical University, Kaohsiung, Taiwan; 7 Department of Biomedical Science and Environmental Biology, Kaohsiung Medical University, Kaohsiung, Taiwan; 8 Interdisciplinary Centre for Natural Products, University of Szeged, Szeged, Hungary; 9 HUN-REN-USZ Biologically Active Natural Products Research Group, University of Szeged, Szeged, Hungary; 10 Department of Oceanography, National Sun Yat-sen University, Kaohsiung, Taiwan; 11 Department of Medical Research, Kaohsiung Medical University Hospital, Kaohsiung, Taiwan; 12 Drug Development and Value Creation Research Center, Kaohsiung Medical University, Kaohsiung, Taiwan

**Keywords:** *Epicoccum sorghinum*, Polyketides, Antimicrobial activity, Fermentation, Non-thermal plasma, Secondary metabolites

## Abstract

*Epicoccum sorghinum* is a filamentous fungus known to produce structurally diverse secondary metabolites. In this study, a fungal strain isolated from *Arundo donax* was investigated for its antimicrobial metabolites and the effects of fermentation conditions and non-thermal atmospheric pressure plasma (NTAPP) treatment on metabolite production. Nine previously reported polyketide-related compounds isolated from *E. sorghinum* were evaluated for their antimicrobial activity against 16 microbial strains. Among the tested compounds, 6-(hydroxymethyl)benzene-1,2,4-triol (7) exhibited the strongest antimicrobial activity among the isolated metabolites, particularly against the Gram-positive bacterium *Staphylococcus epidermidis* ATCC 12228 (MIC = 50 μM). Several compounds showed moderate antifungal activity against *Nakaseomyces glabrata* ATCC 2001. A preliminary structure–activity relationship (SAR) analysis suggested that antimicrobial activity is closely associated with the number and positional arrangement of phenolic hydroxyl groups. Fermentation conditions significantly affected metabolite accumulation. Compound 7 showed significantly higher accumulation under solid fermentation, suggesting that solid fermentation favors the accumulation of certain phenolic-type metabolites. In addition, NTAPP treatment altered the relative abundance of previously detected metabolites in an energy-dependent manner. High-energy treatment (6000 V) increased the abundance of several metabolites, particularly epicorepoxydon A (1), whereas only a slight increase was observed for 7. Overall, this study demonstrates the antimicrobial activity of polyketide-related metabolites from *E. sorghinum* and highlights the influence of fermentation conditions and NTAPP treatment on fungal secondary metabolism. The combination of fermentation optimization and NTAPP stimulation represents a useful strategy for modulating the relative abundance of bioactive secondary metabolites without inducing the production of new detectable metabolites.

## Introduction

Natural products remain an important source of structurally diverse bioactive compounds for drug discovery [1]. Compared with higher plants, microorganisms provide a more sustainable and controllable source of natural products because they can be cultivated under defined conditions and scaled up through fermentation [2, 3]. Consequently, microbial secondary metabolites have become attractive targets for the discovery of biologically active compounds [4].

Among microbial metabolites, polyketide-derived compounds produced by filamentous fungi exhibit a wide range of biological activities, including antimicrobial, anticancer, and anti-inflammatory effects [5-7]. Moreover, sorghum (kaoliang) grain is an important crop with high economic value and diverse applications. In Taiwan, sorghum is widely used in the production of alcoholic beverages, and “Kinmen Kaoliang Liquor” is a well-known brand in Asia. However, fungal contamination represents a major threat to sorghum production, leading to economic losses as well as risks to human and animal health. Several fungal species can infect sorghum grain and produce secondary metabolites, some of which are associated with mycotoxin contamination [8].

*Epicoccum sorghinum* is one of the major fungal contaminants of sorghum grain and has been reported to produce tenuazonic acid (TeA) [9]. However, the strain investigated in this study was isolated from a different host plant, *Arundo donax*. Previous studies have suggested that host-associated interactions and ecological adaptation can influence fungal secondary metabolite biosynthesis. Therefore, isolates originating from different hosts or ecological niches may exhibit distinct metabolic profiles [10]. In addition, *E. sorghinum* produces structurally diverse polyketide metabolites with demonstrated biological activities. In our previous studies, 4,5-dihydroxy-6-(6'-methylsalicyloxy)-2-hydroxymethyl-2-cyclohexen-1-one (**2**) and 6-(hydroxymethyl)benzene-1,2,4-triol (**7**) were identified as major polyketide-related components, showing notable antiangiogenic, antiplatelet, and anti-inflammatory activities [11, 12]. Nevertheless, the antimicrobial potential of these compounds and the metabolic capacity of this species remain insufficiently explored.

In addition, the production of secondary metabolites in microorganisms is strongly influenced by fermentation conditions. Parameters such as temperature, pH, dissolved oxygen, and nutrient composition can significantly affect metabolite yield [13]. Recently, non-thermal atmospheric pressure plasma (NTAPP) has emerged as a controllable stimulation technique capable of generating reactive oxygen and nitrogen species (ROS/RNS), which can modulate cellular signaling pathways and metabolic processes [14]. Previous plasma-related studies have mainly focused on microbial decontamination, mycotoxin reduction, enzyme induction, or plasma-assisted mutagenesis [15-18]. In contrast, the present study investigated the use of NTAPP as a non-mutagenic external stimulus for modulating the relative abundance of previously identified secondary metabolites in *E. sorghinum* [19].

The aim of this study was to investigate the antimicrobial activities of polyketide-related compounds from *E. sorghinum* and to evaluate the effects of different fermentation conditions on metabolite production. Furthermore, NTAPP stimulation was applied to assess its potential for modulating the production of secondary metabolites. This study provides a scientific basis for the antimicrobial potential of polyketide-derived compounds from *E. sorghinum*, and offers new insights into NTAPP-mediated modulation of fungal secondary metabolites.

## Materials and Methods

### Fungal Material

The *E. sorghinum* fungal strain, isolated from *Arundo donax* leaves, was obtained from the Dr. Cecilia Koo Botanic Conservation Center (KBCC), the largest botanical conservation center in Taiwan with over 34322 species of living plants. Its identification was conducted by Ching-Chia Chang and Chi-Ying Li through sequence analysis (GenBank Accession No. KX611667.1) of the internal transcribed spacer (ITS) regions of the rDNA. A voucher specimen with the code number K060107S-B has been archived at the Graduate Institute of Natural Products, College of Pharmacy, Kaohsiung Medical University in Kaohsiung, Taiwan.

### Fermentation and Extraction

The fungal strain *E. sorghinum* underwent two fermentation processes: one on potato dextrose agar (PDA) media and the other on potato dextrose broth (PDB) media. In the first method, the fungus was cultured on PDA and incubated for seven days at 25°C. MeOH extraction was performed on the fermented plates, utilizing a Büchner funnel to separate the solid biomass, followed by concentration of the MeOH extracts under reduced pressure. The extracts underwent partitioning with ethyl acetate (EtOAc) and water, resulting in the isolation of the EtOAc extract (1.55 g). In the second approach, the fungal strain was fermented in PDB media, and the flasks were incubated for seven days on rotatory shakers at 150 rpm and 25°C. After incubation, the fermented broth (48 L) was separated by a Büchner funnel to obtain the filtrate. This filtrate was then subjected to extraction with EtOAc and concentrated under reduced pressure, yielding the EtOAc extract (18.4 g). These two fermentation approaches were employed to compare the secondary metabolite profiles and antimicrobial activities of *E. sorghinum* under solid-state and liquid fermentation conditions.

### Antimicrobial Activity Assay

The test microorganisms comprised standard Gram-positive strains, including *Staphylococcus aureus* ATCC 29213, *Staphylococcus aureus* MRSA 43300, *Staphylococcus epidermidis* ATCC 12228, *Enterococcus faecalis* ATCC 29212, *Streptococcus agalactiae* ATCC 13813, and *Bacillus subtilis* ATCC 6633. The standard Gram-negative strains included *Escherichia coli* ATCC 35218, *Escherichia coli* AG100, *Salmonella Typhimurium* SEO1, *Klebsiella pneumoniae* ATCC 700603, *Pseudomonas aeruginosa* ATCC 27853, and *Moraxella catarrhalis* ATCC 25238. Additionally, the fungal strains employed were *Candida albicans* ATCC 10231, *Candida tropicalis* ATCC 750, *Candida parapsilosis* ATCC 22019, and *Nakaseomyces glabrata* ATCC 2001. Bacterial cultures were cultivated on standard Mueller–Hinton (MH) agar, while fungal cultures were grown on RPMI plates (Diagnosticum Zrt., Hungary) at 37°C overnight in an aerobic environment. Ciprofloxacin and ampicillin (for bacteria) and nystatin (for fungi) were used as positive controls. The minimum inhibitory concentration (MIC) values were determined using the broth microdilution method. Serial two-fold dilutions of purified compounds were prepared over a concentration range of 200–1.56 μM, whereas crude fermentation extracts were prepared over a concentration range of 200–1.56 μg/mL in appropriate culture media. Microbial suspensions were adjusted to approximately 5 × 10^5^ CFU/mL and incubated at 37°C for 16–18 h for bacteria and 24 h for fungi. The fungal incubation conditions were adopted from a previously reported broth microdilution protocol [20] and were applied consistently throughout the study. The MIC was defined as the lowest concentration at which no visible microbial growth was observed.

### HPLC Analysis of Secondary Metabolites

The HPLC system (Shimadzu Inc., Japan) was equipped with an SPD-M10A photodiode array detector and used for all analyses. Separation was achieved using a reversed-phase C18 column (Sunniest C18, 4.6 mm × 250 mm, 5 μm; Nacalai Tesque, Japan), and compounds **1–9** were obtained. The polyketide-related compounds used in this study were previously isolated and structurally characterized from *E. sorghinum* in our earlier study by Chang *et al*. 2020 and 2021 [11, 12]. The names of these compounds are as follows: compound **1**, epicorepoxydon A; compound **2**, 4,5-dihydroxy-6-(6′-methylsalicyloxy)-2-hydroxymethyl-2-cyclohexen-1-one; compound **3**, epicorepoxydon B; compound **4**, 3-hydroxybenzyl alcohol; compound **5**, gentisyl alcohol; compound **6**, hydroxymethyl resorcinol; compound **7**, 6-(hydroxymethyl)benzene-1,2,4-triol; compound **8**, 2-hydroxy-6-methylbenzoic acid; and compound **9**, hydroxytyrosol.

The mobile phases consisted of H_2_O and MeOH. The flow rate was set at 1 mL/min, and the gradient program was as follows: 0–5 min, 10:90 (MeOH:H_2_O); 5–20 min, 10:90 to 30:70; 20–45 min, 30:70 to 0:100. The column was operated at 40°C temperature, and detection was performed at UV 254 nm.

For quantitative analysis, calibration curves were constructed using five different concentrations of each standard compound, and their corresponding HPLC peak areas were recorded. Linearity was assessed by plotting peak area (y, mAU) against concentration (x, g/L). The calibration curves were described by the linear regression equation: y = Ax – B (r^2^ = C), where A represents the slope, B is the intercept, and C is the coefficient of determination (r^2^), indicating the goodness of fit.

The concentrations calculated from the calibration curves were further converted into relative content (%) based on the total weight of the crude extract. The percentage of each compound was calculated by dividing the amount of the compound by the total extract weight and multiplying by 100.

### Non-Thermal Atmospheric Pressure Plasma (NTAPP) Treatment

NTAPP treatment was applied to fungal cultures as an external stimulation strategy. The NTAPP system was powered by an AC high-voltage power supply (PGZ03303-1729-A, You-Shang, Taiwan). Plasma was generated under ambient air conditions at three voltage levels: low (1000 V), medium (3500 V), and high (6000 V). The operating frequency was maintained at approximately 22.22 kHz throughout the experiments. The discharge current varied with the applied voltage and was approximately 32 mA under the 6000 V treatment condition. Because the power supply generated a sinusoidal AC waveform rather than discrete pulses, pulse width was not applicable.

Samples were divided into untreated control and NTAPP-treated groups. Plasma was generated using a needle-electrode system, and the distance between the electrode tip and the sample surface was maintained at 5 mm throughout the experiments. To evaluate the effects of voltage and treatment duration, fungal cultures were exposed to NTAPP under different operating conditions. At 1000 V and 3500 V, samples were treated for 1 and 5 min. Because 6000 V generated a clearly visible and stable plasma plume, further optimization experiments were performed at this voltage using treatment durations of 1, 2, 3, and 4 min. In addition, a 6500 V treatment with a 10 min exposure time was investigated as an exploratory high-energy condition. However, arc discharge frequently occurred at 6500 V, resulting in unstable plasma generation. Therefore, the 6500 V treatment was included only for exploratory evaluation and was not considered during the optimization of NTAPP treatment parameters.

### Heatmap Analysis

Details of the HPLC analysis methods and sample preparation are described above. The effects of different NTAPP treatments on *E. sorghinum* solid fermentation were evaluated by analyzing HPLC peak areas. For each treatment, the peak areas of the detected compounds were normalized to those of the control group (without NTAPP treatment). The normalized values were calculated as the ratio of the peak area of the treated sample to that of the control. All normalized values were compiled into a data matrix and visualized as a heatmap. Values less than 1 were represented in shades of blue, with lower values indicated by a darker blue color. In contrast, values greater than 1 were represented in shades of red, with higher values indicated by a deeper red color. Values equal to 1 were represented as white.

### Statistical Analysis

Quantitative analyses of metabolite contents and NTAPP-induced metabolite abundance at 6000 V for 1, 2, and 3 min were performed using three independent biological replicates (n = 3). Data are presented as mean ± standard deviation (SD). Statistical comparisons between solid and liquid fermentation conditions were performed using Welch's two-tailed t-test. A *p* value < 0.05 was considered statistically significant.

## Results and Discussion

### Antimicrobial Bioactivities Assay

A total of nine polyketide-related compounds were isolated from *E. sorghinum*, and their structures were previously characterized in our earlier studies [11, 12] as shown in Fig. 1. The antimicrobial activities of these compounds and extracts were evaluated against 16 microorganisms, including six Gram-positive bacteria, six Gram-negative bacteria, and four fungal strains. The results are summarized in Tables 1 and 2.

Among all tested compounds, compound **7** exhibited the strongest bioactivity. Regarding antibacterial activity, **7** showed the strongest antibacterial activity against *S. epidermidis* ATCC 12228 (MIC = 50 μM) among the isolated metabolites. It also demonstrated notable activity against *B. subtilis* ATCC 6633 and *M. catarrhalis* ATCC 25238 (MIC = 100 μM), followed by moderate activity against *S. aureus* ATCC 29213 and *S. aureus* MRSA ATCC 43300 (MIC = 200 μM). Compound **5** also exhibited comparable antibacterial activity against *S. epidermidis* ATCC 12228 as well as *M. catarrhalis* ATCC 25238 (MIC = 200 μM).

In terms of antifungal activity, several compounds exhibited moderate activity against *N. glabrata* ATCC 2001. Compounds **2**, **5**, and **7** showed moderate activity (MIC = 100 μM), whereas **1** and **9** showed weak activity, only inhibiting *N. glabrata* at an MIC of 200 μM.

Notably, the extract obtained from solid fermentation exhibited stronger bioactivity than that from liquid fermentation. The solid fermentation extract showed stronger antibacterial activity against *S. epidermidis* ATCC 12228, *S. agalactiae* ATCC 13813, and *M. catarrhalis* ATCC 25238 (MIC = 50 μg/mL), as well as moderate activity against *S. aureus* ATCC 29213, *S. aureus* MRSA 43300, *B. subtilis* ATCC 6633 (MIC = 100 μg/mL), and *E. faecalis* ATCC 29212 (MIC = 200 μg/mL). Furthermore, antifungal activity was also observed against *C. tropicalis* (MIC = 200 μg/mL).

Overall, these results indicate that polyketide-related secondary metabolites from *E. sorghinum*, particularly compounds **2, 5**, and **7**, exhibit measurable antimicrobial activity against selected microorganisms. A consistent trend was observed in which both **7** and the solid fermentation extract exhibited comparatively higher antimicrobial activity than the other tested samples. Notably, several compounds share similar phenolic structures; however, their antimicrobial activities differed considerably, suggesting that subtle structural variations play a critical role in determining bioactivity.

### Structure–Activity Relationships

Similar antimicrobial activities of phenolic polyketide derivatives were reported in previous studies, where hydroxyl-rich aromatic compounds exhibited membrane-disrupting and oxidative stress-inducing properties [21]. Chung *et al*. (2023) reported that compound **5** (gentisyl alcohol), possessing a para-dihydroxy substitution pattern, exhibited moderate antibacterial activity, with minimum inhibitory concentration (MIC) values ranging from 6.25 to 100 μg/mL against various phytopathogenic bacteria [22].

In the present study, compound **7**, which contains a 1,2,4-trihydroxybenzene moiety featuring both ortho- and para-substitution patterns, exhibited stronger antibacterial activity than **5**. This observation suggests that an increased number of hydroxyl groups on the benzene ring may enhance antibacterial activity. In contrast, **6**, characterized by a meta-dihydroxy (resorcinol-type) structure, did not exhibit detectable antibacterial activity. Likewise, **4**, containing only a single hydroxyl group, did not exhibit detectable antibacterial activity. These results indicate that not only the number but also the positional arrangement of hydroxyl groups plays an important role in determining antibacterial activity. This finding is consistent with previous studies, which have emphasized that the substitution pattern of phenolic compounds is a critical factor influencing their biological behavior [21].

Furthermore, compound **9** (hydroxytyrosol), which contains a catechol (ortho-dihydroxy) moiety and has been widely reported to exhibit various biological activities [23], showed only weak antifungal activity in this study, with selective inhibition against *N. glabrata*. This relatively low activity, despite its reported biological activity, may be attributed to structural features such as the presence of a flexible hydroxyethyl side chain.

Overall, although the number of compounds examined in this study is limited, the observed activity trend is consistent with previously reported structure–activity relationships, suggesting that both the number and positional arrangement of hydroxyl groups influence antimicrobial potency. The corresponding SAR schematic is presented in Fig. 2. Compounds such as **7**, which contain multiple hydroxyl substituents on the aromatic ring, exhibited stronger bioactivity, likely due to enhanced redox properties and membrane-disrupting capabilities [24]. Although these compounds differ structurally from classical antifungal agents such as nystatin, a polyketide-derived polyene macrolide, both may exert antifungal effects through disrupting cellular membranes. In contrast to the specific ergosterol-binding mechanism of nystatin, these phenolic compounds likely induce membrane damage through oxidative stress and nonspecific interactions with lipid bilayers [25].

### HPLC Fingerprint Analysis (Qualitative)

Following the antimicrobial evaluation, we observed a consistent trend in which both the solid fermentation extract and compound **7** exhibited comparatively higher antimicrobial activity than the other tested samples. Although liquid fermentation is generally considered more suitable for rapid growth and large-scale production [26], it is of interest to investigate the differences in metabolite profiles between solid and liquid fermentation and the relationship between compound abundance and bioactivity. Therefore, HPLC fingerprinting was performed to qualitatively analyze the metabolite profiles of polyketide-related compounds under different fermentation conditions.

*E. sorghinum* was cultured using both solid-state and liquid fermentation. The HPLC-UV fingerprints of the extracts are presented in Fig. 3. The chromatographic profiles revealed notable differences between solid and liquid fermentation. Although the same set of compounds was detected under both conditions and no new peaks were observed, the relative peak intensities varied considerably between the two conditions. Moreover, clear differences in peak areas were observed, suggesting substantial variation in metabolite abundance. All compounds were detectable under both fermentation conditions, although some appeared only at very low intensities. Compounds **1** and **7** were the dominant metabolites in solid fermentation, whereas compound **2** was more abundant in liquid fermentation. Compounds **5** and **9** showed relatively low peak intensities in both solid and liquid fermentation extracts despite exhibiting notable antimicrobial activity. The higher abundance of **7** in the solid fermentation extract may contribute to its comparatively stronger antimicrobial activity. In addition, **7** was one of the major metabolites detected in the solid fermentation HPLC profile and exhibited the strongest antimicrobial activity among the isolated metabolites. These observations suggest that **7** may play an important role in the bioactivity of the solid fermentation extract. The complete HPLC–UV fingerprints of the extracts are provided in the Supporting Information (Fig. S1).

### Quantitative Analysis of Metabolites under Different Fermentation Conditions

Based on the qualitative HPLC fingerprint analysis, quantitative analysis was further performed on the major peaks to allow accurate comparison of metabolite levels. Compounds **1**, **2**, **7**, and **8** were quantified under the established HPLC conditions using their corresponding calibration curves. The results of the quantitative analysis are summarized in Table 3, and detailed calibration curve data are provided in the Supporting Information (Fig. S2). Compound **1** was quantified using the calibration equation: y = 5.8696 x − 0.3201 (r^2^ = 0.9965) to determine its concentration in both solid and liquid fermentation. The content of **1** was 0.62 ± 0.19% in solid fermentation and 0.50 ± 0.15% in liquid fermentation. Compound **2** was quantified using the calibration equation: y = 7.7827 x − 0.2509 (r^2^ = 0.9993). The content of **2** was 0.25 ± 0.08% in solid fermentation and 4.72 ± 0.46% in liquid fermentation. Compound **7** was quantified using the calibration equation: y = 0.5566 x + 0.0118 (r^2^ = 0.9998). The content of **7** was 7.64 ± 1.39% in solid fermentation and 1.54 ± 0.69% in liquid fermentation. Compound **8** was quantified using the calibration equation: y = 8.599 x + 0.07 (r^2^ = 0.9966). The content of **8** was 1.74 ± 1.61% in solid fermentation and 0.41 ± 0.23% in liquid fermentation. Raw quantitative data used for the calculation of mean ± SD values are provided in the Supporting Information (Table S3).

Overall, compound **7** exhibited the highest mean content in solid fermentation (7.64 ± 1.39%), whereas compound **2** showed the highest mean content in liquid fermentation (4.72 ± 0.46%). These findings demonstrate that fermentation conditions play a critical role in influencing secondary metabolite accumulation. Solid fermentation tended to favor the accumulation of certain phenolic-type metabolites, including **7**, whereas liquid fermentation favored the production of other metabolites such as **2**. This observation is consistent with previous studies showing that different fermentation modes (solid-state vs submerged) can significantly alter the composition and distribution of secondary metabolites produced by fungi [27].

### External Stimulation by Non-thermal Atmospheric Pressure Plasma

Based on the results above, compound **7** was identified as the most active antimicrobial compound among the isolated metabolites, and solid fermentation was selected as the optimal condition to increase its production. Non-thermal atmospheric pressure plasma (NTAPP) has been reported to enhance metabolite production in plants [28]. This effect is generally attributed to NTAPP-generated reactive oxygen and nitrogen species (ROS/RNS), which can induce oxidative stress and thereby activate biosynthetic pathways [14]. Therefore, NTAPP was applied as an external stimulation strategy.

The effect of NTAPP on metabolite accumulation was evaluated using HPLC profiling. The complete HPLC profiles under different plasma energy conditions are provided in the Supporting Information (Figs. S3–S6). The results were recorded and presented as a heatmap (Fig. 4). The detailed raw data, treatment conditions, and numbers of experimental replicates are provided in Tables S1 and S2. Heatmap analysis revealed that NTAPP treatment altered the relative abundance of previously detected metabolites in an energy-dependent manner. Under the 6000 V, 1 min condition, compound **1** showed the greatest increase in relative abundance (2.45 ± 2.11), whereas compounds **2** and **7** exhibited moderate increases (1.56 ± 0.31 and 1.15 ± 0.16, respectively) compared with the untreated control. Detailed quantitative data are provided in Table S4. These findings indicate that NTAPP stimulation did not selectively enhance the accumulation of the most bioactive compound, as only a modest increase was observed for **7** under the investigated conditions.

This observation suggests that NTAPP can act as an effective external stimulus to modulate secondary metabolism in *E. sorghinum*. Although NTAPP treatment altered the accumulation of several previously detected metabolites, the biological consequences of these metabolic changes remain unclear.

Importantly, NTAPP treatment did not result in the appearance of new detectable metabolites but instead altered the relative abundance of metabolites already produced by *E. sorghinum*. These results indicate modulation of existing metabolic pathways rather than activation of previously silent or cryptic biosynthetic pathways. Future studies should further investigate the relationship between NTAPP-induced metabolic changes and biological activity and optimize NTAPP parameters to selectively influence the production of target metabolites.

## Conclusion

In this study, nine polyketide-related compounds were isolated from *E. sorghinum* and evaluated for their antimicrobial activities. Among them, 6-(hydroxymethyl)benzene-1,2,4-triol (**7**) exhibited the strongest antimicrobial activity, likely due to the presence of multiple phenolic hydroxyl groups. Structure–activity relationship (SAR) analysis indicated that both the number and positional arrangement of hydroxyl groups are key factors influencing antimicrobial potency.

Fermentation conditions significantly affected metabolite accumulation. Solid fermentation favored the accumulation of certain phenolic-type metabolites, including compound **7**. In addition, non-thermal atmospheric pressure plasma (NTAPP) modulated the accumulation of previously detected secondary metabolites. Under the investigated conditions, NTAPP treatment produced the greatest increase in epicorepoxydon A (**1**), whereas only a modest increase was observed for **7**.

Overall, this study demonstrates the antimicrobial potential of polyketide-related metabolites from *E. sorghinum*. The combination of fermentation optimization and NTAPP stimulation represents a promising strategy for modulating fungal secondary metabolism and the relative abundance of bioactive metabolites. Furthermore, compound **7** exhibited the strongest antimicrobial activity among the isolated metabolites and warrants further investigation, including evaluation of its cytotoxicity, selectivity, and safety profiles.

## Supplemental Materials

Supplementary data for this paper are available on-line only at http://jmb.or.kr.



## Figures and Tables

**Fig. 1:**
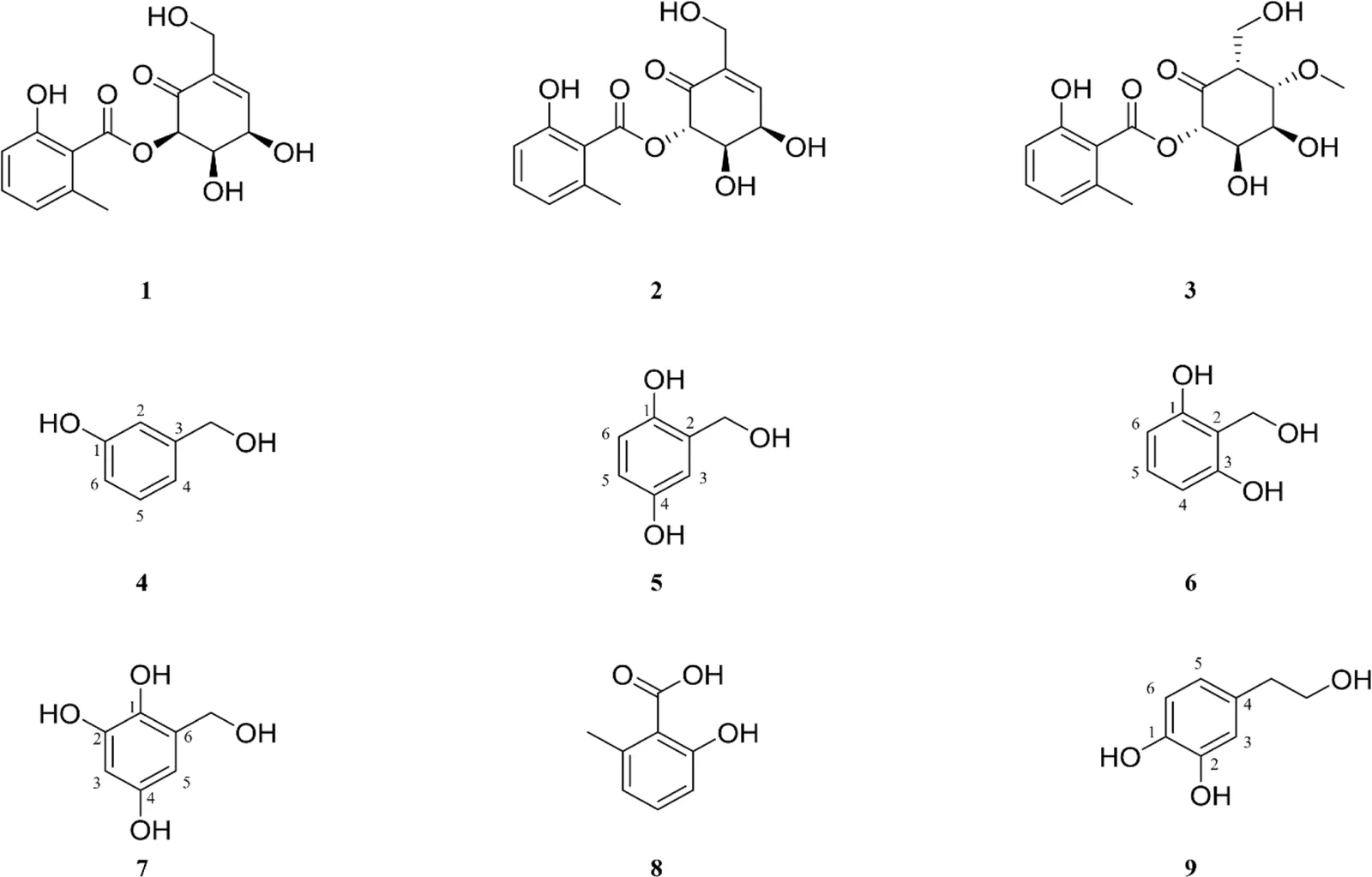
Chemical structures of compounds 1–9 isolated from *Epicoccum sorghinum* (previously reported compounds).

**Fig. 2:**
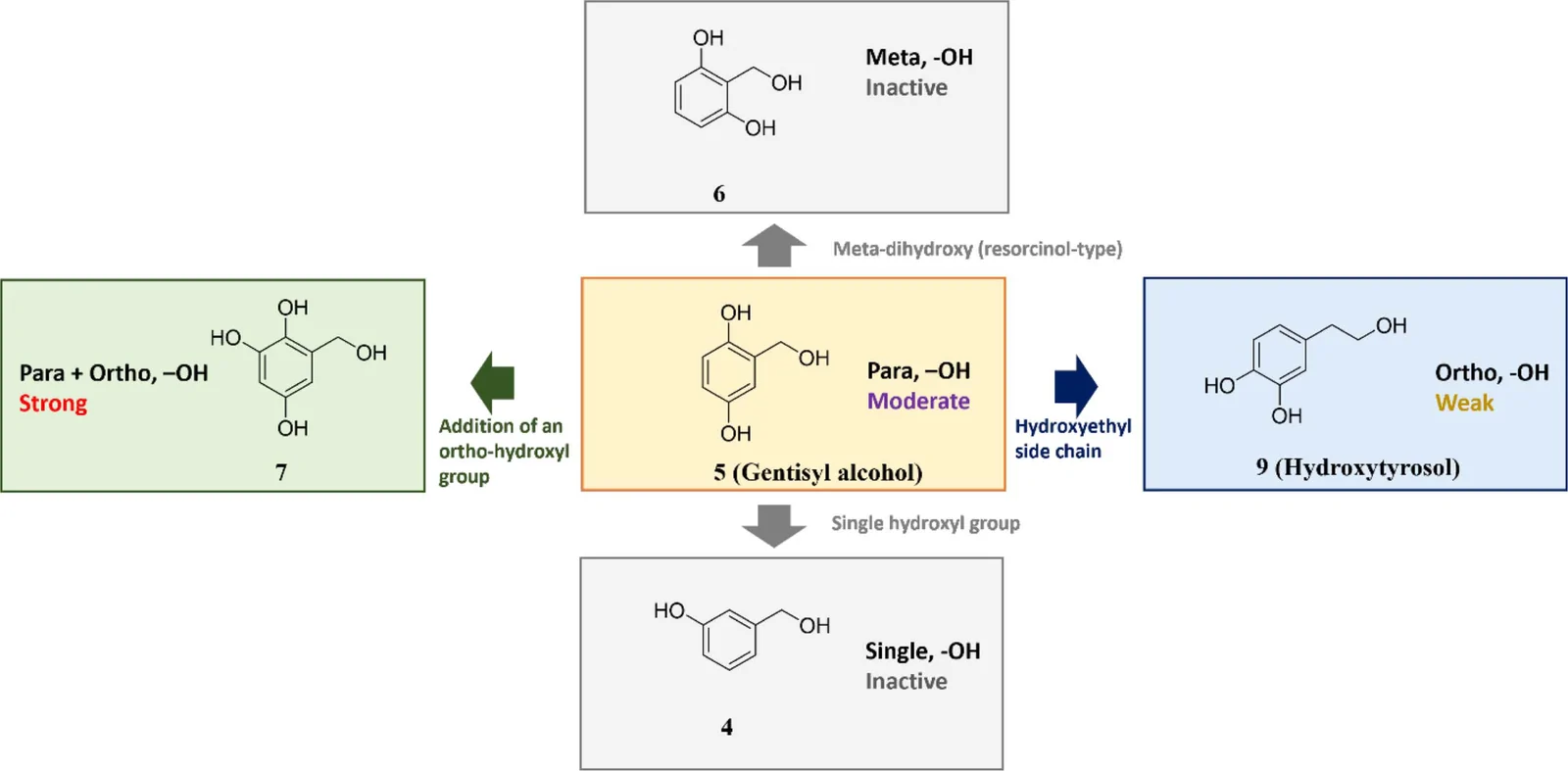
Structure–activity relationships of polyketide-derived antimicrobial metabolites from *Epicoccum sorghinum*. Both the number and positional arrangement of hydroxyl groups may influence antimicrobial potency.

**Fig. 3:**
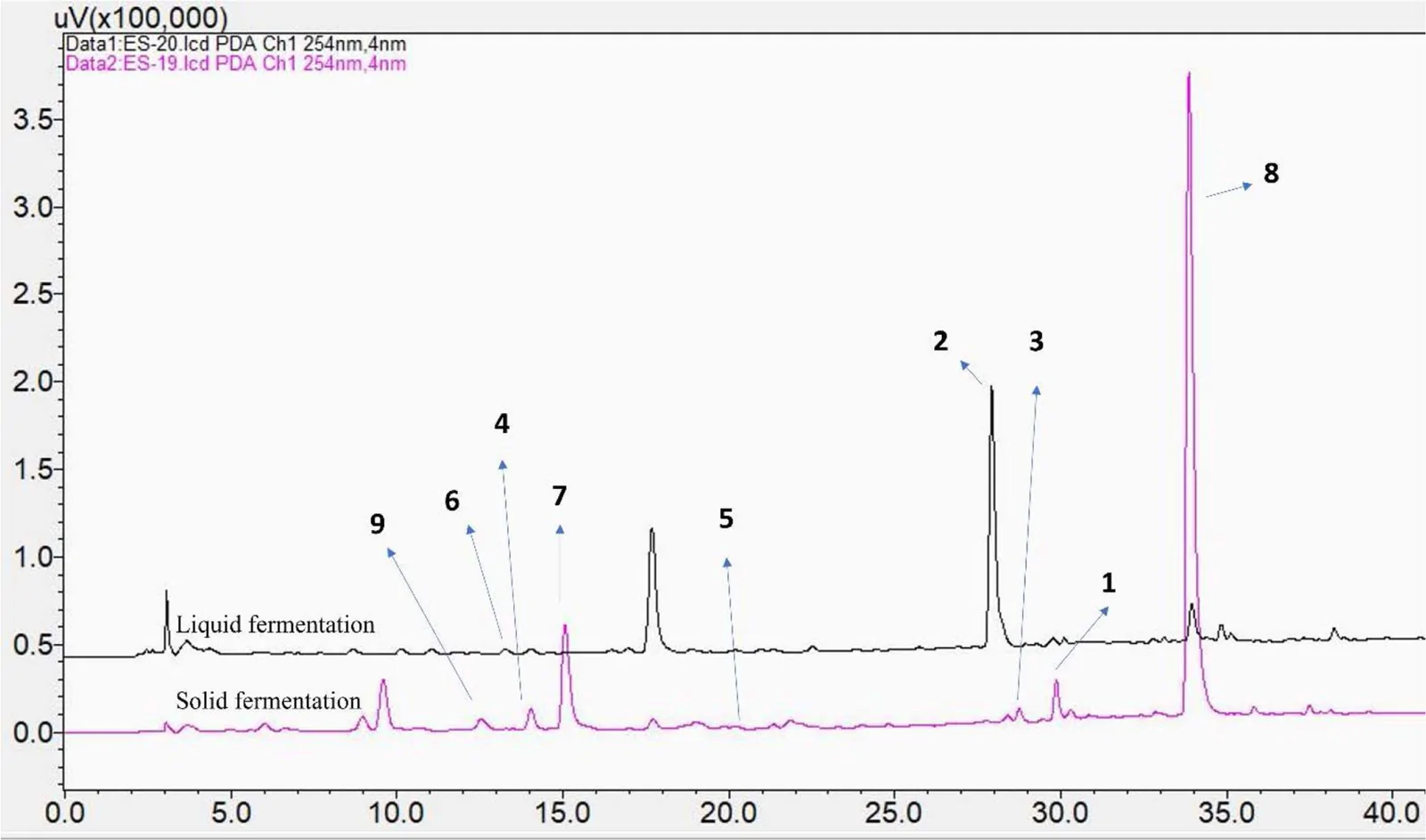
HPLC fingerprint of *Epicoccum sorghinum* extracts obtained from solid and liquid fermentation. All polyketide-related compounds were detected in the HPLC fingerprint under a gradient elution system (MeOH–H2O, 5–100% over 60 min). Notably, compound 7 exhibited a prominent peak in extracts derived from solid fermentation, whereas compound 2 showed a more pronounced peak in liquid fermentation.

**Fig. 4:**
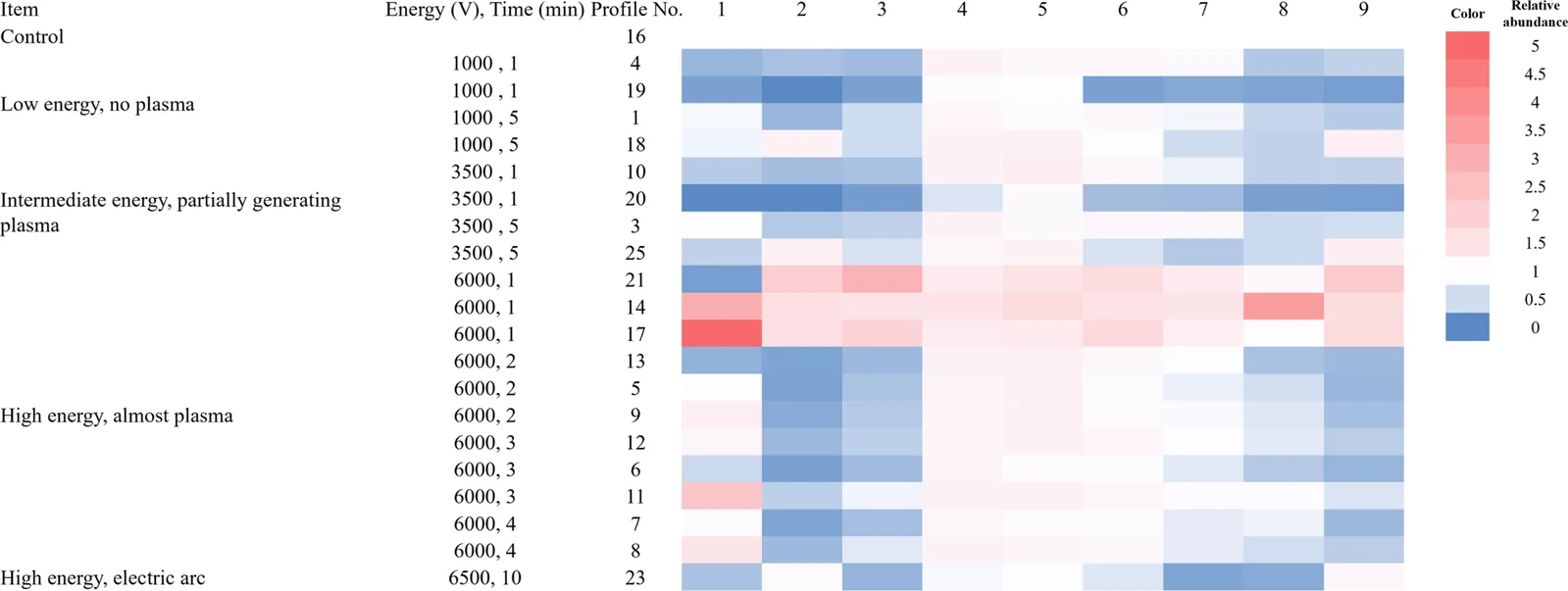
Heatmap analysis of changes in the relative abundance of metabolites under different NTAPP treatment conditions. Heatma*p* values represent normalized peak-area ratios relative to the untreated control (set as 1.0). High-energy and prolonged NTAPP treatment generally reduced the relative abundance of several detected metabolites. The 6000 V, 1 min treatment resulted in increased relative abundance of several metabolites, particularly compound 1. Compounds 2 and 7 exhibited slight upward trends following NTAPP treatment. Data were obtained from independent biological replicates, and the raw normalized values are provided in Table S2.

**Table 1:** Antimicrobial activities of compounds 1–9 isolated from *Epicoccum sorghinum*.

		Gram-positive bacteria					
		*S. aureus* ATCC 29213	*S. aureus* MRSA 43300	*S. epidermidis* ATCC 12228	*E. faecalis* ATCC 29212	*S. agalactiae* ATCC 13813	*B. subtilis* ATCC 6633
MIC(μM)	1	>200	>200	>200	>200	>200	>200
	2	>200	>200	>200	>200	>200	>200
	3	>200	>200	>200	>200	>200	>200
	4	>200	>200	>200	>200	>200	>200
	5	>200	>200	**200**	>200	>200	>200
	6	>200	>200	>200	>200	>200	>200
	7	**200**	**200**	**50**	>200	>200	**100**
	8	>200	>200	>200	>200	>200	>200
	9	>200	>200	>200	>200	>200	>200
	Ciprofloxacin	0.3125	0.625	0.3125	1.56	1.56	0.0625
	Ampicillin	0.78	12.5	3.125	1.56	0.0625	0.03125
		Gram-negative bacteria					
		*E. coli* ATCC 35218	*E. coli* AG100	*S.* Typhimurium SEO1	*K. pneumoniae* ATCC 700603	*P. aeruginosa* ATCC 27853	*M. catarrhalis* ATCC 25238
MIC(μM)	1	>200	>200	>200	>200	>200	>200
	2	>200	>200	>200	>200	>200	>200
	3	>200	>200	>200	>200	>200	>200
	4	>200	>200	>200	>200	>200	>200
	5	>200	>200	>200	>200	>200	**200**
	6	>200	>200	>200	>200	>200	>200
	7	>200	>200	>200	>200	>200	**100**
	8	>200	>200	>200	>200	>200	>200
	9	>200	>200	>200	>200	>200	>200
	Ciprofloxacin	0.019	0.039	0.0625	0.39	0.195	0.0625
	Ampicillin	>500	6.25	1.56	>500	500	0.0078
		Fungi					
		*C. albicans* ATCC 10231	*C.tropicalis* ATCC 750	*C. parapsilosis* ATCC 22019	*N. glabrata* ATCC 2001		
MIC(μM)	1	>200	>200	>200	**200**		
	2	>200	>200	>200	**100**		
	3	>200	>200	>200	>200		
	4	>200	>200	>200	>200		
	5	>200	>200	>200	**100**		
	6	>200	>200	>200	>200		
	7	>200	>200	>200	**100**		
	8	>200	>200	>200	>200		
	9	>200	>200	>200	**200**		
	Nystatin	0.625	0.625	0.3125	0.3125		

**Table 2:** Antimicrobial activities of solid- and liquid-fermentation extracts from *Epicoccum sorghinum*.

		Gram-positive bacteria					
		*S. aureus* ATCC 29213	*S. aureus* MRSA 43300	*S. epidermidis* ATCC 12228	*E. faecalis* ATCC *29212*	*S. agalactiae* ATCC 13813	*B. subtilis* ATCC 6633
MIC(μg/mL)	Solid fermentation	**100**	**100**	**50**	**200**	**50**	**100**
	Liquid fermentation	>200	>200	>200	>200	>200	>200
	Ciprofloxacin	0.104	0.207	0.104	0.517	0.517	0.021
	Ampicillin	0.273	4.368	1.092	0.545	0.022	0.011
		Gram-negative bacteria					
		*E. coli* ATCC 35218	*E. coli* AG100	*S.* Typhimurium SEO1	*K. pneumoniae* ATCC 700603	*P. aeruginosa* ATCC 27853	*M. catarrhalis* ATCC 25238
MIC(μg/mL)	Solid fermentation	>200	>200	>200	>200	>200	**50**
	Liquid fermentation	>200	>200	>200	>200	>200	>200
	Ciprofloxacin	0.006	0.013	0.021	0.129	0.065	0.021
	Ampicillin	>174.7	2.184	0.545	>174.7	174.7	0.0027
		Fungi					
		*C. albicans* ATCC 10231	*C.tropicalis* ATCC 750	*C. parapsilosis* ATCC 22019	*N. glabrata* ATCC 2001		
MIC(μg/mL)	Solid fermentation	>200	**200**	>200	**50**		
	Liquid fermentation	**200**	>200	>200	>200		
	Nystatin	0.579	0.579	0.289	0.289		

**Table 3:** Contents of major compounds (1, 2, 7, and 8) under different fermentation conditions.

Compounds	Content (%) in solid fermentation	Content (%) in liquid fermentation	*p*-value
1	0.62 ± 0.19	0.50 ± 0.15	0.438
2	0.25 ± 0.08	4.72 ± 0.46	0.003
7	7.64 ± 1.39	1.54 ± 0.69	0.007
8	1.74 ± 1.61	0.41 ± 0.23	0.288

Note: Data are expressed as mean ± SD from three independent fermentation experiments (n = 3). *p*-values were calculated using Welch’s two-tailed t-test to compare compound contents between solid and liquid fermentation. A *p*-value < 0.05 was considered statistically significant.
