# Supplementary material for: Polyketide-Derived Antimicrobial Metabolites from Epicoccum sorghinum: Effects of Fermentation Conditions and Non-Thermal Atmospheric Pressure Plasma (NTAPP)
Source: J Microbiol Biotechnol. 2026 Oct 13;36:e2606052. doi: 10.4014/jmb.2606.06052 (PMC13697207; doi:10.4014/jmb.2606.06052)
Supplement: Supplementary file 1 [file jmb-36-e2606052-supple.pdf]

J. Microbiol. Biotechnol. 2026. 36: e2606052
https://doi.org/10.4014/jmb.2606.06052

Supplementary Figures and Tables

Polyketide-Derived Antimicrobial Metabolites from *Epicoccum sorghinum*: Effects of Fermentation Conditions and Non-Thermal Atmospheric Pressure Plasma (NTAPP)

Ching-Chia Chang [a, †], Bai-Wei Peng [a, b, †], Annamária Kincses [c, i], Chi-Ying Li [d], Hua-Han Chen [e], Hsueh-Wei Chang [a, f, g], Judit Hohmann [c, h, i,*], Fang-Rong Chang [a, j, k, l,*]

[a] *Graduate Institute of Natural Products, College of Pharmacy, Kaohsiung Medical University, Kaohsiung, Taiwan*
[b] *Pharmacognosy, Department of Pharmaceutical Biosciences, Biomedical Centre, Uppsala, Sweden*
[c] *Institute of Pharmacognosy, University of Szeged, Szeged, Hungary*
[d] *School of Pharmacy, Tzu Chi University, Hualien, Taiwan*
[e] *Department of Food Science, National Penghu University of Science and Technology, Makung City, Penghu Hsien, Taiwan*
[f] *Cancer Center, Kaohsiung Medical University Hospital, Kaohsiung Medical University, Kaohsiung, Taiwan*
[g] *Department of Biomedical Science and Environmental Biology, Kaohsiung Medical University, Kaohsiung, Taiwan*
[h] *Interdisciplinary Centre for Natural Products, University of Szeged, Szeged, Hungary*
[i] *HUN-REN-USZ Biologically Active Natural Products Research Group, University of Szeged, Szeged, Hungary*
[j] *Department of Oceanography, National Sun Yat-sen University, Kaohsiung, Taiwan*
[k] *Department of Medical Research, Kaohsiung Medical University Hospital, Kaohsiung, Taiwan*
[l] *Drug Development and Value Creation Research Center, Kaohsiung Medical University, Kaohsiung, Taiwan*

* Corresponding authors

* Judit Hohmann, hohmann.judit@szte.hu

* Fang-Rong Chang, aaronfrc@kmu.edu.tw

Contents

Fig. S1. HPLC–UV chromatograms of extracts obtained from solid and liquid fermentation.

Fig. S2. Calibration curves of major metabolites.

Fig. S3. HPLC chromatograms of *E. sorghinum* extracts from solid fermentation without NTAPP treatment (control).

Fig. S4. HPLC chromatograms of *E. sorghinum* extracts under low-energy NTAPP treatment.

Fig. S5. HPLC chromatograms of *E. sorghinum* extracts under intermediate-energy NTAPP treatment.

Fig. S6. HPLC chromatograms of *E. sorghinum* extracts under high-energy NTAPP treatment.

Table S1. Experimental design of NTAPP treatments and corresponding HPLC profiles.

Table S2. Raw normalized relative-abundance values used for heatmap construction under different NTAPP treatment conditions.

Table S3. Raw quantitative data for fermentation comparison.

Table S4. Relative abundance of metabolites under NTAPP treatment at 6000 V for 1, 2, and 3 min.

The supporting information contains additional HPLC chromatograms, calibration curves, raw quantitative data, and normalized abundance values used for data analysis and heatmap construction.

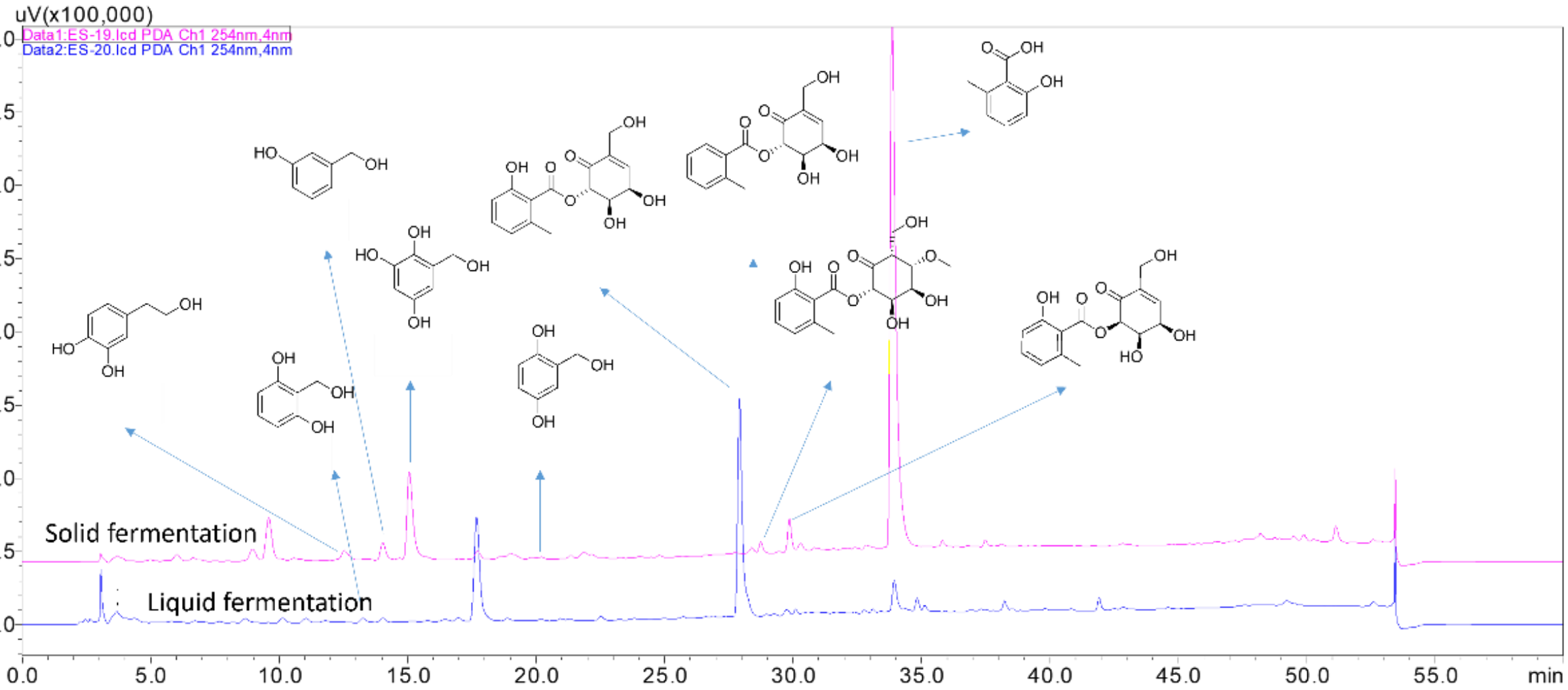

Fig. S1. HPLC–UV chromatograms of extracts obtained from solid and liquid fermentation.

Compound 1

Concentration (g/L)	Peak Area (MAU)
1.0000	5.622831
0.5000	2.529501
0.2500	0.973488
0.1250	0.433062
0.0625	0.213223

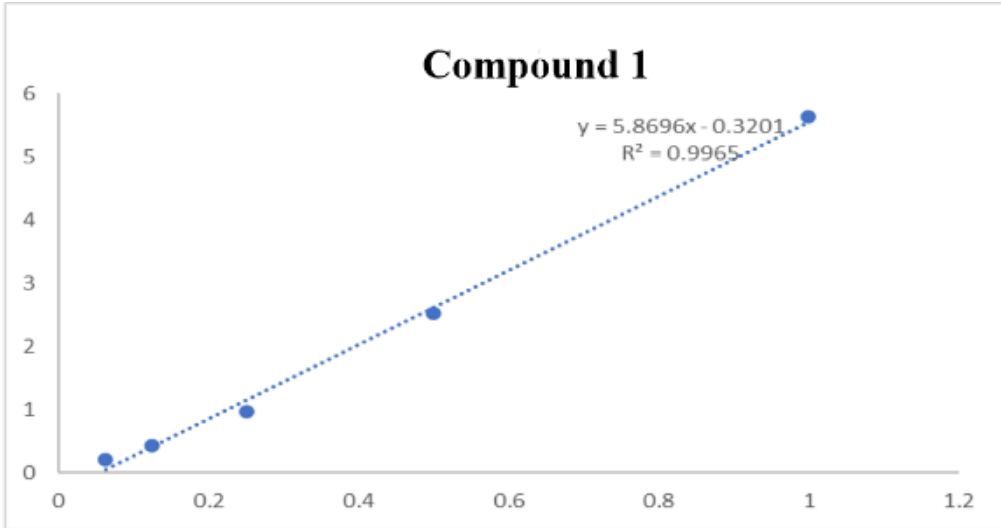

Compound 2

Concentration (g/L)	Peak Area (MAU)
1.0000	7.587019
0.5000	3.548632
0.2500	1.627669
0.1250	0.73841
0.0625	0.322815

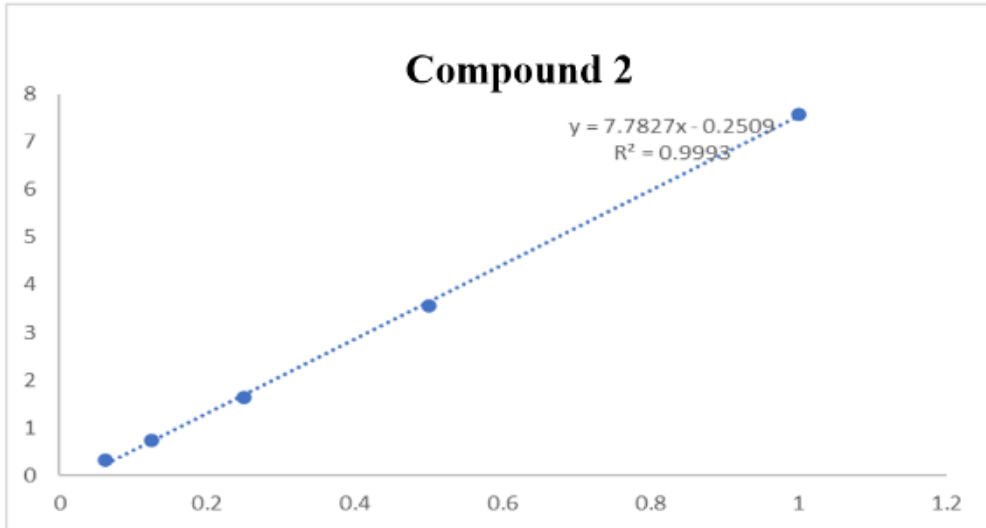

Compound 7

Concentration (g/L)	Peak Area (MAU)
1.0000	0.566272
0.5000	0.293492
0.2500	0.15449
0.1250	0.080901
0.0625	0.042566

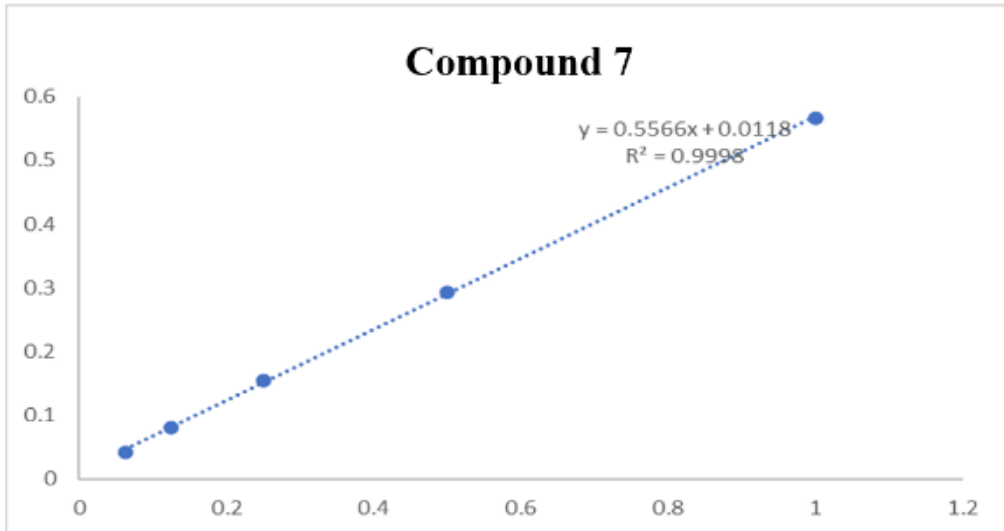

Compound 8

Concentration (g/L)	Peak Area (MAU)
1.0000	8.519585
0.5000	4.658669
0.2500	2.309291
0.1250	1.09329
0.0625	0.429663

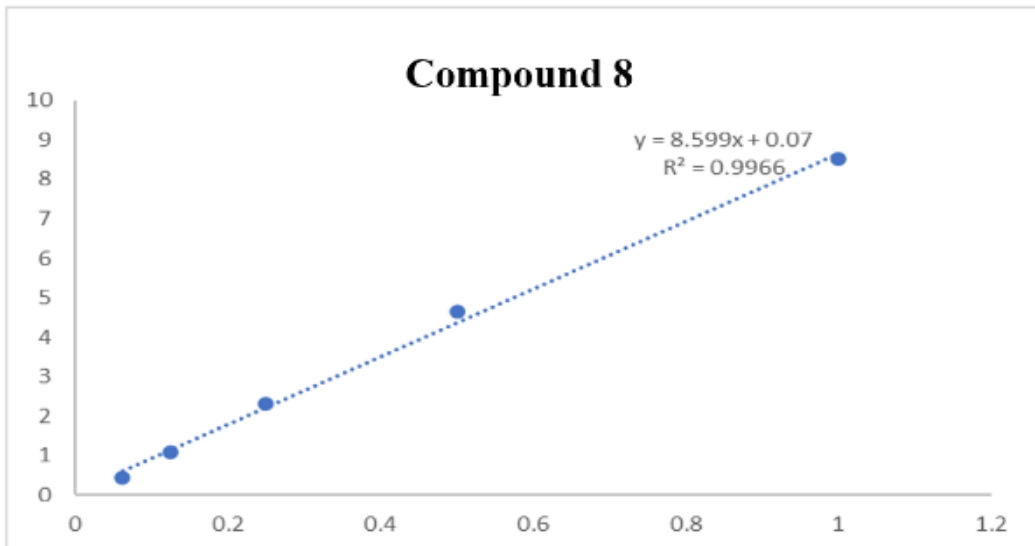

Fig. S2. Calibration curves of major metabolites.

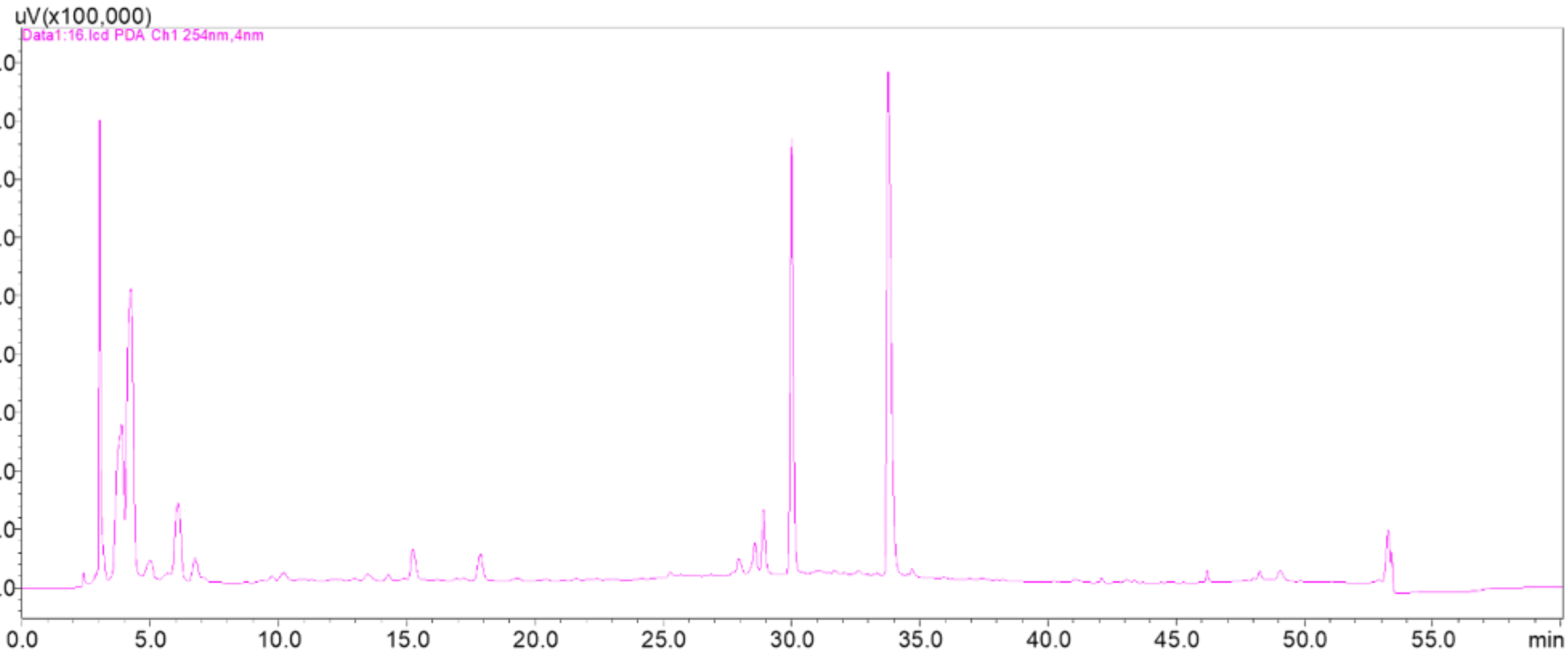

Fig. S3. HPLC chromatograms of *E. sorghinum* extracts from solid fermentation without NTAPP treatment (control).

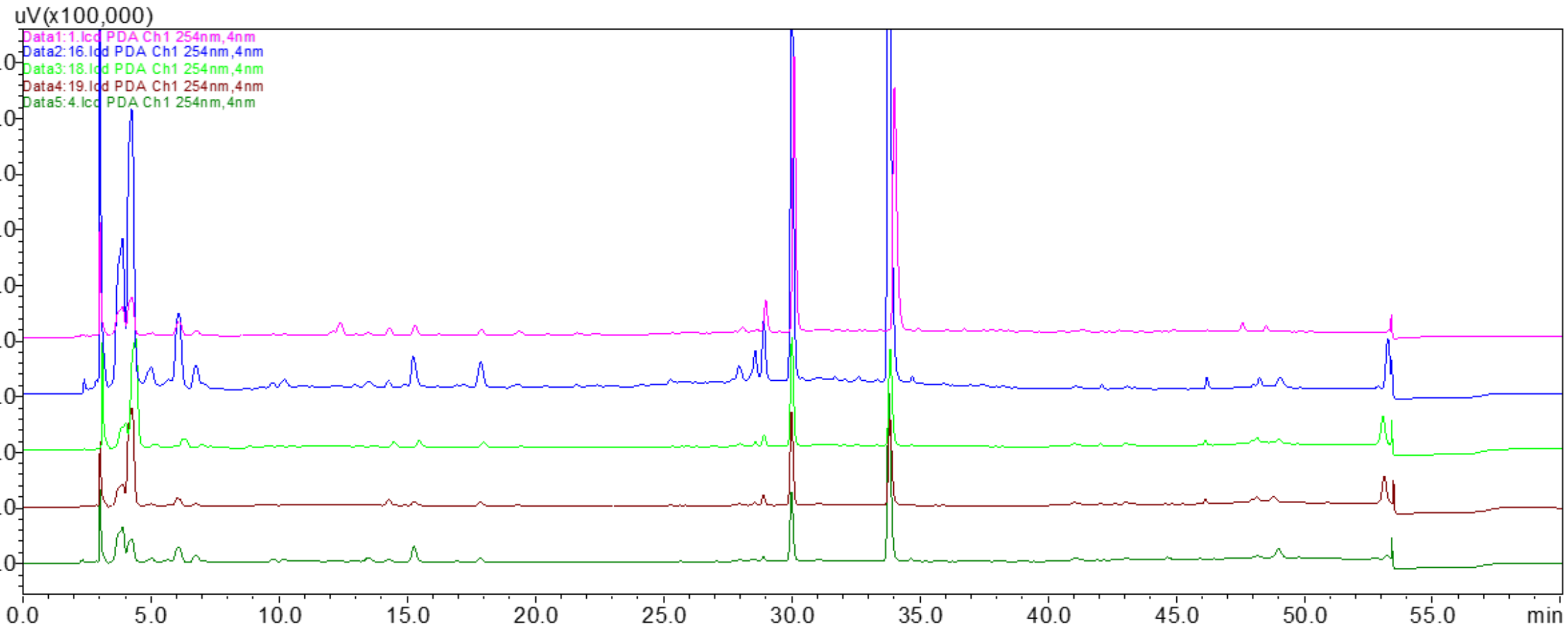

Fig. S4. HPLC chromatograms of *E. sorghinum* extracts under low-energy NTAPP treatment.

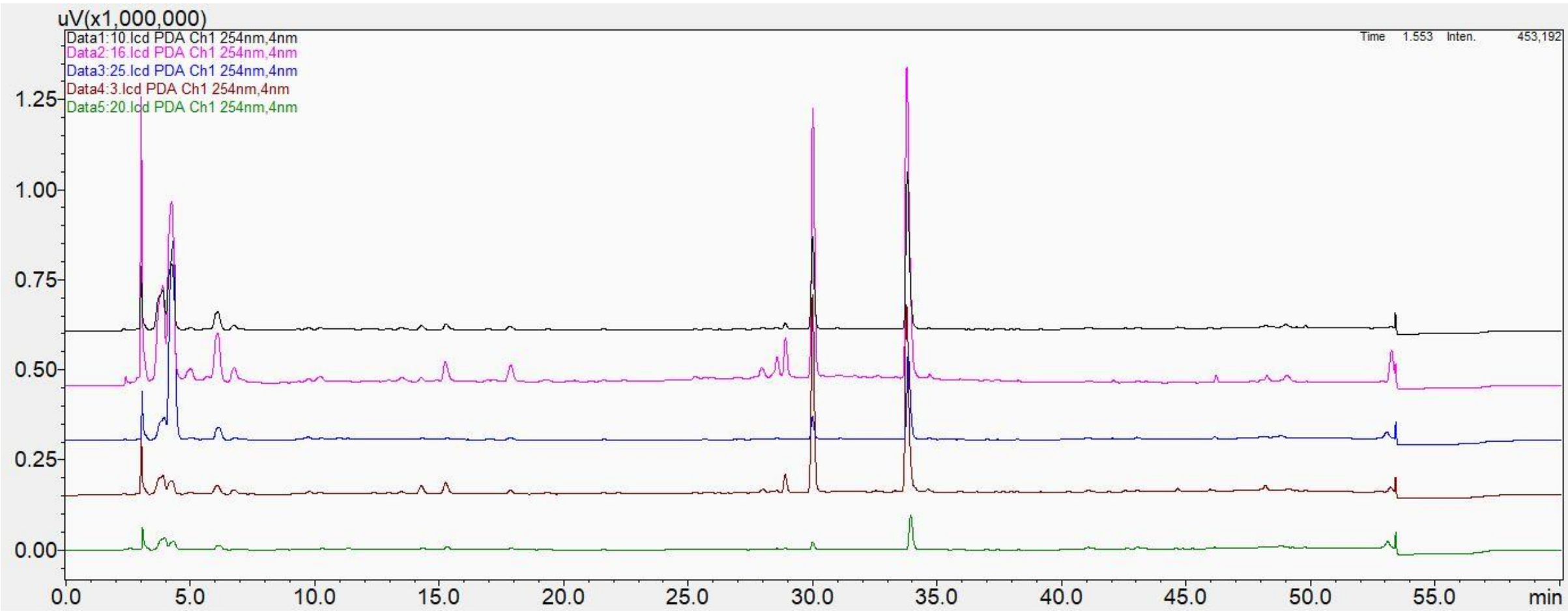

Fig. S5. HPLC chromatograms of *E. sorghinum* extracts under intermediate-energy NTAPP treatment.

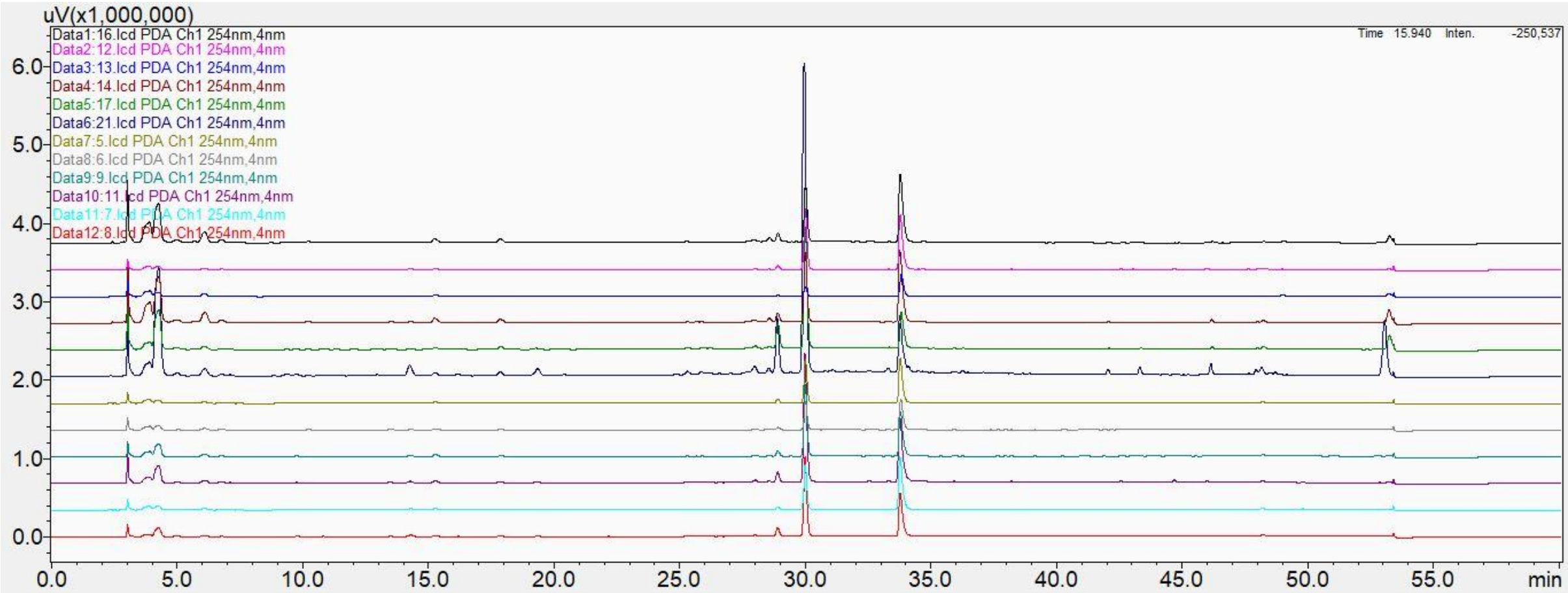

Fig. S6. HPLC chromatograms of *E. sorghinum* extracts under high-energy NTAPP treatment.

Table S1. Experimental design of NTAPP treatments and corresponding HPLC profiles.

Treatment Category	Voltage (V)	Time (min)	Profile No.	Replicates (n)
Control	0	0	16	1
Low energy, no plasma	1000	1	4, 19	2
Low energy, no plasma	1000	5	1, 18	2
Intermediate energy	3500	1	10, 20	2
Intermediate energy	3500	5	3, 25	2
High energy, almost plasma	6000	1	21, 14, 17	3
High energy, almost plasma	6000	2	13, 5, 9	3
High energy, almost plasma	6000	3	12, 6, 11	3
High energy, almost plasma	6000	4	7, 8	2
High energy, electric arc	6500	10	23	1

Table S2. Raw normalized relative-abundance values used for heatmap construction under different NTAPP treatment conditions.

Item	Energy (V), Time (min)	Profile No.	**1**	**2**	**3**	**4**	**5**	**6**	**7**	**8**	**9**
Control		16	1	1	1	1	1	1	1	1	1
Low energy, no plasma	1000 , 1	4	0.33	0.39	0.36	0.94	0.8	0.8	0.76	0.42	0.47
	1000 , 1	19	0.23	0.12	0.23	0.72	0.7	0.23	0.27	0.25	0.22
	1000 , 5	1	0.66	0.34	0.52	0.87	0.74	0.81	0.65	0.49	0.44
	1000 , 5	18	0.64	0.91	0.52	0.93	0.98	0.7	0.52	0.47	1.01
Intermediate energy, partially generating plasma	3500 , 1	10	0.44	0.37	0.39	0.93	1.07	0.79	0.63	0.47	0.47
	3500 , 1	20	0.12	0.12	0.21	0.56	0.77	0.37	0.36	0.23	0.22
	3500 , 5	3	0.68	0.43	0.47	0.91	0.76	0.84	0.78	0.51	0.53
	3500 , 5	25	0.47	0.96	0.55	0.86	0.93	0.55	0.43	0.51	1.06
High energy, almost plasma	6000, 1	21	0.22	1.86	2.58	1.14	1.35	1.53	1.14	0.79	1.96
	6000, 1	14	2.69	1.39	1.29	1.32	1.52	1.34	1.3	3.16	1.49
	6000, 1	17	4.44	1.42	1.71	1.1	1.14	1.61	1.01	0.69	1.52
	6000, 2	13	0.31	0.25	0.35	0.98	0.99	0.8	0.69	0.39	0.35
	6000, 2	5	0.7	0.24	0.4	0.9	0.92	0.74	0.62	0.53	0.34
	6000, 2	9	1.03	0.28	0.43	0.88	0.96	0.75	0.67	0.57	0.38
	6000, 3	12	0.87	0.35	0.46	0.89	0.92	0.84	0.68	0.59	0.45
	6000, 3	6	0.51	0.23	0.36	0.89	0.75	0.71	0.58	0.43	0.33
	6000, 3	11	2.1	0.46	0.64	0.94	0.99	0.85	0.75	0.71	0.56
	6000, 4	7	0.73	0.25	0.38	0.87	0.75	0.72	0.6	0.63	0.35
	6000, 4	8	1.31	0.35	0.58	0.91	0.9	0.79	0.6	0.53	0.45
High energy, electric arc	6500, 10	23	0.39	0.73	0.32	0.66	0.69	0.57	0.25	0.28	0.83

Note: Values represent normalized peak-area ratios relative to the untreated control. The untreated control was assigned a value of 1.0. For conditions with multiple profile numbers, each profile represents an independent biological replicate.

Table S3. Raw quantitative data for fermentation comparison.

Replicate	Compound 1 (Solid, %)	Compound 1 (Liquid, %)	Compound 2 (Solid, %)	Compound 2 (Liquid, %)	Compound 7 (Solid, %)	Compound 7 (Liquid, %)	Compound 8 (Solid, %)	Compound 8 (Liquid, %)
R1	0.52	0.33	0.19	4.20	9.20	1.30	1.35	0.16
R2	0.84	0.58	0.34	5.05	7.15	1.00	3.50	0.61
R3	0.51	0.60	0.22	4.91	6.56	2.32	0.36	0.46

Note: R1–R3 represent three independent fermentation experiments. Metabolite contents (%) were determined by HPLC using external calibration curves and are expressed as the percentage of each compound relative to the total crude extract weight.

Table S4. Relative abundance of metabolites under NTAPP treatment at 6000 V for 1, 2, and 3 min.

	6000 V, 1 min (n = 3)	6000 V, 2 min (n = 3)	6000 V, 3 min (n = 3)
**Compound**	**Mean ± SD**		
**1**	2.45 ± 2.11	0.68 ± 0.36	1.16 ± 0.85
**2**	1.56 ± 0.31	0.26 ± 0.02	0.35 ± 0.12
**3**	1.86 ± 0.66	0.39 ± 0.03	0.49 ± 0.14
**4**	1.19 ± 0.12	0.92 ± 0.05	0.91 ± 0.03
**5**	1.34 ± 0.19	0.96 ± 0.03	0.89 ± 0.12
**6**	1.49 ± 0.14	0.76 ± 0.03	0.80 ± 0.07
**7**	1.15 ± 0.16	0.66 ± 0.03	0.67 ± 0.09
**8**	1.55 ± 1.39	0.50 ± 0.09	0.58 ± 0.14
**9**	1.66 ± 0.28	0.36 ± 0.02	0.45 ± 0.12

Note: Values are expressed as mean ± SD from three independent biological replicates (n = 3). Relative abundance values were normalized to the untreated control, which was assigned a value of 1.0.
